# Hepcidin predicts response to IV iron therapy in patients admitted to the intensive care unit: a nested cohort study

**DOI:** 10.1186/s40560-018-0328-2

**Published:** 2018-09-10

**Authors:** Edward Litton, Stuart Baker, Wendy Erber, Shannon Farmer, Janet Ferrier, Craig French, Joel Gummer, David Hawkins, Alisa Higgins, Axel Hofmann, Bart De Keulenaer, Julie McMorrow, John K. Olynyk, Toby Richards, Simon Towler, Robert Trengove, Steve Webb, on behalf of the IRONMAN Study investigators, Andy Chapman, Elizabeth Jenkinson, Anne Marie Palermo, Brigit Roberts, the Australian and New Zealand Intensive Care Society Clinical Trials Group

**Affiliations:** 1 Intensive Care Unit, Fiona Stanley Hospital, Perth, Western Australia 6065 Australia; 2 School of Medicine, University of Western Australia, Perth, Western Australia 6009 Australia; 3 Intensive Care Unit, Sir Charles Gardner Hospital, Perth, Western Australia 6009 Australia; 4 School of Patholody, University of Australia, Perth, Western Australia 6009 Australia; 5 Medical School, Faculty of Health and Medical Sciences, University of Western Australia, Perth, Western Australia 6009 Australia; 6 Western Health, Melbourne, Victoria Australia; 7 University of Melbourne, Melbourne, Victoria Australia; 8 Separation Science and Metabolomics Laboratory Metabolomics Australia (Western Australia node), Murdoch University, Perth, Western Australia Australia; 9 Intensive Care Unit, Joondalup Health Campus, Joondalup, Western Australia Australia; 10 Centre of Research Excellence for Patient Blood Management in Critical Illness and Trauma, Monash University, Melbourne, Victoria Australia; 11 Intensive Care Unit, Royal Perth Hospital, Perth, Western Australia 6000 Australia; 12 School of Medicine, University of Western Australia, Perth, Western Australia 6009 Australia; 13 University College London, London, UK; 14 Intensive Care Unit, Fiona Stanley Hospital, Perth, Western Australia 6150 Australia

**Keywords:** Anaemia, Critical care, Hepcidin, Intravenous iron, Red blood cell transfusion

## Abstract

**Background:**

Both anaemia and red blood cell (RBC) transfusion are common and associated with adverse outcomes in patients admitted to the intensive care unit (ICU). The aim of this study was to determine whether serum hepcidin concentration, measured early after ICU admission in patients with anaemia, could identify a group in whom intravenous (IV) iron therapy decreased the subsequent RBC transfusion requirement.

**Methods:**

We conducted a prospective observational study nested within a multicenter randomized controlled trial (RCT) of IV iron versus placebo. The study was conducted in the ICUs of four tertiary hospitals in Perth, Western Australia. Critically ill patients with haemoglobin (Hb) of < 100 g/L and within 48 h of admission to the ICU were eligible for participation after enrolment in the IRONMAN RCT. The response to IV iron therapy compared with placebo was assessed according to tertile of hepcidin concentration.

**Results:**

Hepcidin concentration was measured within 48 h of ICU admission in 133 patients. For patients in the lower two tertiles of hepcidin concentration (< 53.0 μg), IV iron therapy compared with placebo was associated with a significant decrease in RBC transfusion requirement [risk ratio 0.48 (95% CI 0.26–0.85), *p* = 0.013].

**Conclusions:**

In critically ill patients with anaemia admitted to an ICU, baseline hepcidin concentration predicts RBC transfusion requirement and is able to identify a group of patients in whom IV iron compared with placebo is associated with a significant decrease in RBC transfusion requirement.

**Trial registration:**

Australian New Zealand Clinical Trials Registry: ANZCTRN12612001249 Registered 26/11/2012

**Electronic supplementary material:**

The online version of this article (10.1186/s40560-018-0328-2) contains supplementary material, which is available to authorized users.

## Background

Anaemia is common in patients admitted to the intensive care unit (ICU) and is associated with adverse outcomes [1, 2]. Despite evidence to support a restrictive red blood cell (RBC) transfusion threshold, anaemia is also the most common indication for RBC transfusion in the ICU, itself associated with increased morbidity and mortality [3].

In non-critically ill patients, intravenous (IV) iron therapy is effective in promoting erythropoiesis and decreasing RBC transfusion requirement [4]. However, recent randomized controlled trials (RCTs) of IV iron therapy in critically ill patients have not demonstrated benefit [5, 6]. This may be because critical illness results in an acute inflammatory response that confounds the interpretation of standard, clinically available measures of iron deficiency used in these studies. By contrast, the serum concentration of hepcidin, a key regulator of iron metabolism, decreases in response to iron-restricted erythropoiesis, even in the presence of inflammation [7]. Hepcidin is a small peptide secreted predominantly by the liver and acts by blocking duodenal iron absorption and decreasing availability of iron stored in hepatocytes and macrophages to red cell precursors. Although hepcidin may be more accurate in predicting an increase in red blood cell production in response to IV iron, prospective clinical data is lacking.

The primary aim of this study was to determine whether low serum hepcidin concentration could identify a subset of critically ill patients with anaemia in whom IV iron therapy was effective in reducing RBC transfusion requirement.

## Methods

The study was a prospective cohort study, nested within the IRONMAN RCT, the protocol and primary results of which have previously been published [5, 8]. Briefly, the IRONMAN RCT enrolled adult patients who were within 48 h of admission to ICU, had a hemoglobin (Hb) of less than 100 g/L, and were anticipated to require ICU care beyond the next calendar day. Exclusion criteria included suspected or confirmed severe sepsis, a ferritin greater than 1200 ng/mL or transferrin saturation greater than 50%. Participants were randomised in a 1:1 ratio to receive either 500 mg IV ferric carboxymaltose or placebo and were followed up to hospital discharge. Human Research Ethics Committee approval was obtained at all sites prior to commencement, and prospective consent was obtained from all participants or their legal surrogates.

For this nested cohort study, blood was collected for serum hepcidin measurement immediately following enrolment and prior to study drug administration. Hepcidin-25 was isolated from blood for quantitation by liquid chromatography-quadrupole time-of-flight mass spectrometry (LC-qTOF-MS), using a Waters Synapt G2S (Waters, Manchester, UK) as previously described [9, 10]. Hepcidin was isolated by solid phase extraction following the initial addition of a synthetic human hepcidin (^13^C_18_,^15^N_3_) peptide internal standard (Peptides International, Kentucky, USA) and removal of the more abundant polypeptides by organic solvent precipitation and centrifugation. The accurate mass measurement of the precursor hepcidin-25 [M+5H]^5+^ ion was confirmed against a hepcidin-25 standard (Peptides International, Kentucky, USA) and further by MS/MS. Quantitation was by reference to a human hepcidin-25 (^13^C_18_,^15^N_3_) calibration, prepared in human serum.

The primary objective was to determine whether hepcidin concentration could identify a group of critically ill patients for whom IV iron therapy was effective in decreasing the risk of RBC transfusion. The secondary objective was to develop a prognostic model for RBC transfusion quantity in patients admitted to the ICU.

### Statistical analysis

Continuous variables were reported as mean (± SD) or median and interquartile range (IQR), with between-group differences analyzed using Student’s *t* test or the Wilcoxon rank-sum test for apparently normal and non-normally distributed data respectively. Categorical variables were reported as proportion and analyzed using the *χ*^2^ test or Fischer exact test as appropriate. Data was censored at 60 days after enrolment for RBC transfusion Hb concentration and vital status.

The relationship between hepcidin concentration and response to IV iron therapy was examined using similar methodology to a previously published RCT of IV iron in patients with chemotherapy-induced anaemia conducted by Steensma et al. [11]. Similar to this methodology, patients were first stratified by tertile of baseline hepcidin concentration then combined. The incident risk ratio for RBC transfusion was then compared between those who were randomized to receive IV iron and those who received placebo. The relationship between IV iron therapy and RBC transfusion quantity across the range of hepcidin values was further explored by locally weighted scatterplot smoothing (LOWESS) [12]. Receiver-operator characteristic curve analysis was not undertaken because the IRONMAN RCT demonstrated nearly identical overall proportions of patients transfused in the two groups.

The model to predict subsequent RBC transfusion quantity in patients admitted to the ICU was developed using negative binomial univariate and multivariate analyses. Variables with a *p* value of < 0.3 on univariate analysis were included in a multivariable analysis with backwards selection with an alpha of 0.05. Interaction was assessed using multivariable fractional polynomials to account for potential non-linear relationships. Significant interactions (*p* value of < 0.05) were examined graphically, and a final model was then produced. The relative prognostic value with and without baseline hepcidin concentration included was assessed using Akaike information criterion (AIC).

Outcome data was censored at 60 days after enrolment. A two-sided *p* value of 0.05 or less was considered to be statistically significant. All analyses were conducted with Stata Version 14 StataCorp College Station, TX77845, USA.

## Results

**Fig. 1:**
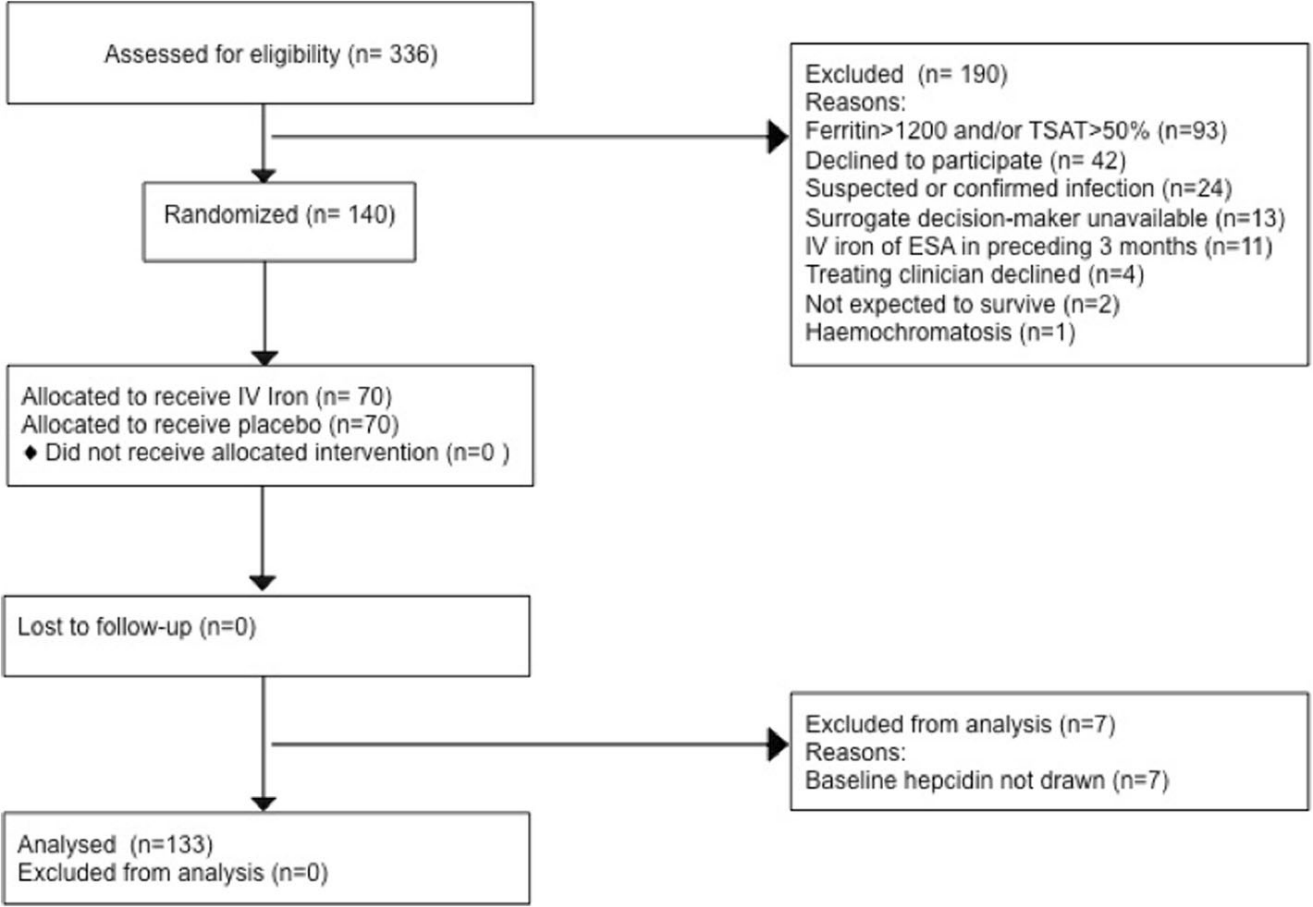
Derivation of the cohort

Baseline hepcidin levels were available for 133 (95%) out of the 140 participants enrolled in the IRONMAN RCT. The flow of participants is presented in Fig. 1. The mean time from ICU admission to collection was 29 h [standard deviation (SD) 13], and median hepcidin concentration was 34.9 μg/L [interquartile range (IQR) 17.3–69.2, range 0–163.5]. The baseline characteristics of the population are provided in Table 1. There was no significant correlation between hepcidin concentration and baseline C reactive protein or iron indices (see Additional file 1).

**Table 1:** Baseline characteristics

Characteristic^*^	Outcome (*n* = 133)
Age—years	62 (41–73)
Male gender—no. (%)	91 (68)
ICU admission source—no (%)	
Emergency department	24 (18)
Hospital ward	8 (6)
Operating theater	97 (73)
Other hospital	4 (3)
ICU admission type—no (%)	
Medical	17 (13)
General surgical	20 (15)
Cardiothoracic	49 (37)
Trauma	42 (32)
Neurosurgical	5 (4)
APACHE II score	12 (9–17)
SOFA score	6 (4–9)
Prior RBC transfusion—no (%)	30 (23)
Haemoglobin—g/L	88 (81–94)
Mean corpuscular volume—fL	91 (88–94)
C reactive protein—mg/L	110 (48–170)
Iron—mcg/dL	3 (2–6)
Ferritin—ng/ml^+^	260 (161–437)
Transferrin—mg/dL	17 (15–20)
Transferrin saturation—%	9 (6–16)
Soluble transferrin receptor—mg/L	1.81 (1.28–2.44)
Hepcidin—μg/mL	34.9 (17.3–69.2)
Tertile 1—(0–20.08)	10.6 (4.2–15.6)
Tertile 2—(20.09–53.00)	34.9 (27.1–48.5)
Tertile 3—(53.01–163.46)	81.2 (69.2–97.9)

*ICU* intensive care unit, *APACHE* acute physiology and chronic health evaluation, *SOFA* sequential organ failure assessment, *RBC* red blood cell

^*^Median and interquartile range (IQR) unless otherwise reported

^+^ng/ml has a conversion factor of 1 to the standard international units mcg/ml

### Hepcidin and prediction of response to IV iron

**Fig. 2:**
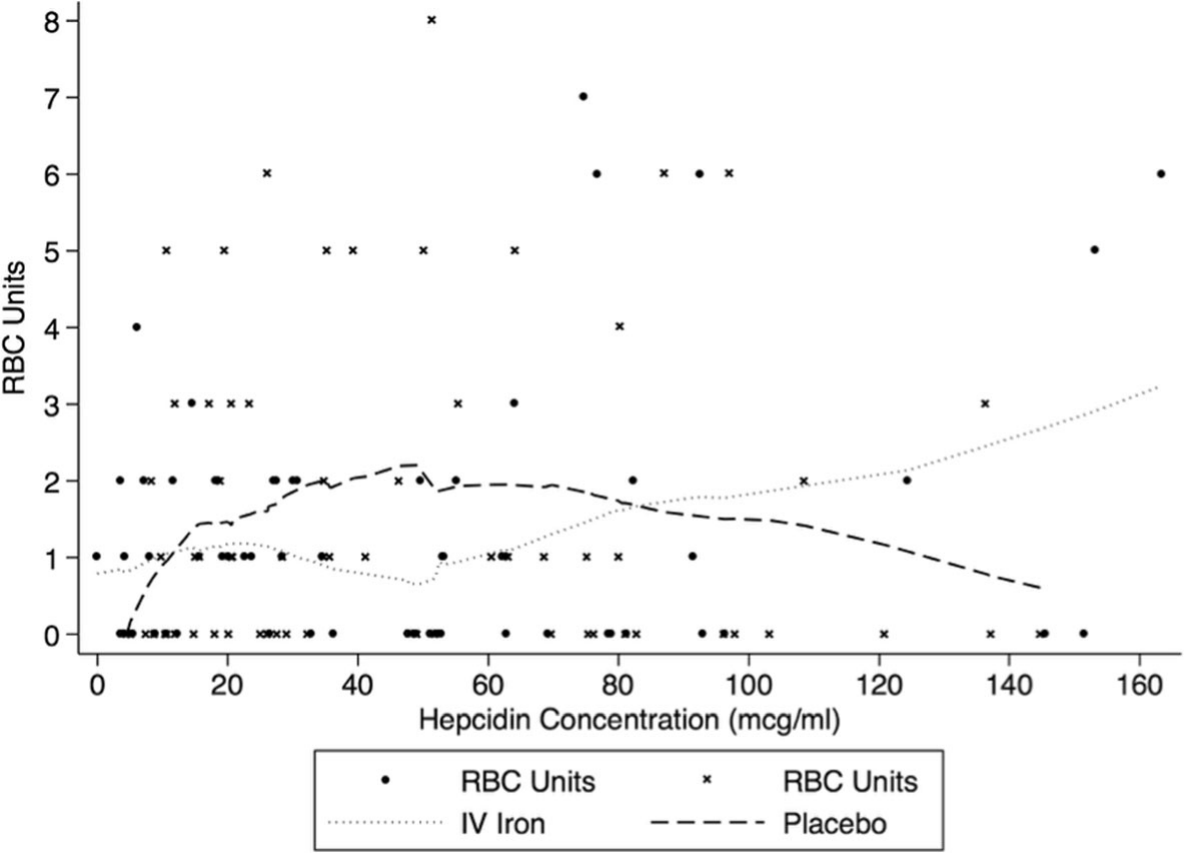
Association between hepcidin concentration and Red Blood Cell units transfused for patients receiving IV iron and placebo

Of the 88 patients in the lower two tertiles of hepcidin concentration (0 to 53.0 μg/L), 44 received IV iron therapy and 44 received placebo. In patients with a low hepcidin concentration (≤ 53.0 μg/L), the relative risk (RR) of RBC transfusion associated with IV iron was 0.48 (95% CI 0.26–0.85), *p* = 0.013. In patients with a high hepcidin level (> 53.0 μg/L), there was no significant association between IV iron therapy and risk of RBC transfusion, RR 1.33 (95% CI 0.57–3.08), *p* = 0.518. The association between IV iron therapy and RBC transfusion by tertile of hepcidin concentration is provided in Table 2. The association between hepcidin concentration and RBC transfusion quantity in patients who received IV iron compared with placebo is represented by the LOWESS plot in Fig. 2.

**Table 2:** Hepcidin and risk of RBC transfusion with IV iron therapy

Variable	IV iron	Placebo	Risk ratio (95% CI)	*p* value
Hepcidin ≤ 53.0 μg/L				
Number RBC units/patients	38/44	80/44		
Median RBC units (IQR)	1 (0–2)	1 (0–3)	0.48 (0.26–0.85)	0.013
Hepcidin 1st tertile (0–20.1 μg/L)				
Number RBC units/number patients	23/22	35/21		
Median RBC units (IQR)	1 (0–2)	0 (0–2)	0.63 (0.26–1.50)	0.293
Hepcidin 2nd tertile (20.1–53.0 μg/L)				
Number RBC units/number patients	15/22	45/23		
Median RBC units (IQR)	0 (0–1)	1 (0–3)	0.35 (0.16–0.77)	0.009
Hepcidin 3rd tertile (> 53.0 μg/L)				
Number RBC units/number patients	43/22	34/23		
Median RBC units (IQR)	1 (0–3)	1 (0–3)	1.33 (0.57–3.08)	0.518

*RBC* red blood cell, *IQR* interquartile range

IV iron therapy compared with placebo was not associated with a significant increase in Hb for those in the lower two tertiles of hepcidin values [mean increase Hb 3 g/L (95% CI − 3–10), *p* = 0.361]. There was no significant correlation between hepcidin and concurrent Hb, ferritin, transferrin saturation, soluble transferrin receptor or C reactive protein. There was also no significant difference in iron, transferrin saturation, ferritin or transferrin receptor levels associated with lower versus higher hepcidin tertile (see Additional file 1).

### Predicting RBC transfusion quantity

The complete list of variables assessed on univariate analysis and those added to the initial multivariable model is provided in Additional file 1. ICU admission related to trauma, baseline Hb < 80 g/L, lower transferrin saturation and lower hepcidin concentration were found to predict increased risk of RBC transfusion and were retained in the final multivariable model. There was a significant interaction between Hb and hepcidin concentrations in predicting the risk of RBC transfusion (likelihood ratio test for significance of interaction *p* = 0.0462), see Additional file 1: Figure S1. For patients with a Hb ≥ 80 g/L, each 10 μg/mL increase in hepcidin concentration was associated with a risk ratio of RBC transfusion of 1.09 (95% CI 1.01–1.18, *p* = 0.034). However, for patients with a Hb < 80 g/L, there was no significant association between hepcidin concentration and risk of RBC transfusion [RR 0.95 (95% CI 0.84–1.07), *p* = 0.387]. The variables included in the final model are provided in Table 3. AIC with hepcidin in the model was 415.14 versus 468.16 with hepcidin removed.

**Table 3:** Final multivariate model—independent predictors of RBC transfusion

Characteristic (*n* = 133)	Coefficient (95% CI)	Risk ratio (95% CI)	*p* value
ICU admission type—trauma vs non trauma	0.833 (0.382–1.285)	2.30 (1.46–3.61)	< 0.001
Haemoglobin > 80 g/L^*^—yes vs no	− 0.99 (− 1.493 to − 0.493)	0.37 (0.22–0.61)	< 0.001
Transferrin saturation—per 10% increase	0.237 (0.082–0.391)	1.27 (1.09–1.48)	0.003
Hepcidin—per 10 μg/ml increase	0.086 (0.030–0.142)	1.09 (1.03–1.15)	0.002

*CI* confidence interval, *ICU* intensive care unit. Constant for model 0.088 (95% CI − 0.398–0.575)

^*^Likelihood ratio test for significance of interaction between haemoglobin as a continuous variable and hepcidin concentration in predicting the risk of RBC transfusion *p* = 0.0462. For patients with a haemoglobin < 80 g/L, there was no significant association between hepcidin concentration and risk of RBC transfusion [RR 0.95 (95% CI 0.84–1.07), *p* = 0.387]

## Discussion

We found that serum hepcidin concentration identified a subset of anaemic, critically ill patients in whom IV iron therapy was effective in reducing RBC transfusion requirement. These findings are important when considering the dose-response relationship between increasing RBC transfusion quantity and worse clinical outcomes and also due to the scarcity and cost of RBC transfusion. In addition to predicting the effectiveness of IV iron in critically ill patients, we also found that hepcidin concentration was an independent predictor of RBC transfusion requirement but that the association was modified by Hb levels.

Although Lasocki et al. have described low hepcidin concentrations in some critically ill patients with anaemia, there is limited data exploring whether hepcidin can be used to guide treatment decisions [7]. Steensma et al. found that hepcidin concentration could predict response to IV iron therapy in patients with chemotherapy-induced anaemia [11]. In our study, the median C reactive protein was 110 mg/L but did not correlate with hepcidin concentration, suggesting that even in the presence of inflammation, measurement of hepcidin is useful in identifying critically ill patients in whom IV iron therapy is likely to reduce RBC transfusion requirement [13]. Our findings also support further investigation into the role of hepcidin antagonists in patients with elevated hepcidin [14].

Hepcidin synthesis is finely regulated and induced by both inflammation and iron overload [15]. Hepcidin levels have been shown to be the predominant predictor of erythrocyte iron incorporation in African children with anaemia [16]. Amongst adult patients admitted to the ICU, Tacke et al. have demonstrated an association between markers of increased iron availability and mortality [17]. It is plausible that using hepcidin concentration to target IV iron therapy in critically ill patients can also reduce the potential risk of initiating or exacerbating infection associated with both excessively high free iron levels and iron deficiency.

Accurate prediction of RBC transfusion quantity could provide an additional method to target IV iron therapy in critically ill patients. In the IRONMAN RCT, subgroup analysis did not suggest a differential effect of IV iron related to transferrin saturation or ferritin concentration [5]. Building on these findings, the current study found no significant difference in iron indices based on hepcidin tertile or correlation between iron indices and hepcidin. Although iron-restricted erythropoiesis is not present in all critical patients who require RBC transfusion, our study found that hepcidin was an independent predictor of subsequent transfusion. Although our model requires validation, a metric of observed versus predicted RBC transfusion may also be useful as a quality metric.

Future studies must also address the substantial variation that currently exists in hepcidin assays and reference ranges that makes comparison between studies difficult [14]. A reliable point of care hepcidin test is necessary to provide results in a clinically useful timeframe. The ongoing HEPCIDANE RCT (NCT02276690) of hepcidin-guided management of anaemia in critically ill patients will address some of these issues.

### Limitations

Our study was limited to patients with an Hb < 100 g/L and did not enroll patients with sepsis. Whether hepcidin measurement is useful in these patients remains uncertain. Although soluble transferrin receptor levels were not found not to be predictive of RBC transfusion requirement in our study, other assays with potential diagnostic benefit including zinc protophoryn were not assessed. However, given the central role of hepcidin in iron metabolism, it is unlikely that other assays provide superior diagnostic utility. The relatively small number of participants precluded the use of methods to reduce the risk of over-fitting of the statistical models, and larger studies are required to validate the findings. Finally, the current study only assessed the use of hepcidin at a single point in time. Greater understanding is required of how hepcidin levels change over time including the effect interventions such as RBC transfusion and IV iron therapy.

## Conclusion

In patients with anaemia admitted to the ICU, hepcidin measurement can identify a group of patients in whom IV iron therapy decreases RBC transfusion requirement.

## Additional file

Additional file 1: **Figure S1.** Relationship between hepcidin concentration and RBC transfusion, moderated by haemoglobin concentration. Hb Haemoglobin, RBC red blood cell. **Table S1.** Iron indices according to hepcidin levels. **Table S2.** Univariate analysis of variables associated with risk of RBC transfusion. CI confidence interval, ICU intensive care unit, APACHE acute physiology and chronic health evaluation, SOFA sequential organ failure assessment. Variables in bold added to the initial multivariable model. (DOCX 108 kb)

## Abbreviations

CI: Confidence interval
Hb: Haemoglobin
ICU: Intensive care unit
IQR: Interquartile range
IV: Intravenous
RBC: Red blood cell
RCT: Randomized controlled trial
RR: Risk ratio
SD: Standard deviation
