# Supplementary material for: Hepcidin predicts response to IV iron therapy in patients admitted to the intensive care unit: a nested cohort study
Source: J Intensive Care. 2018 Sep 10;6:60. doi: 10.1186/s40560-018-0328-2 (PMC6131742; doi:10.1186/s40560-018-0328-2)
Supplement: Supplementary file 1 — Figure S1. Relationship between hepcidin concentration and RBC transfusion, moderated by haemoglobin concentration. Hb Haemoglobin, RBC red blood cell. Table S1. Iron indices according to hepcidin levels. Table S2. Univariate analysis of variables associated with risk of RBC transfusion. CI confidence interval, ICU intensive care unit, APACHE acute physiology and chronic health evaluation, SOFA sequential organ failure assessment. Variables in bold added to the initial multivariable model. (DOCX 108 kb) [file 40560_2018_328_MOESM1_ESM.docx]

Additional file 1

1 – Complete Eligibility Criteria for the IRONMAN RCT

## Inclusion criteria

1. Admitted to an ICU for less than 48 hours
2. Anticipated to require ICU care beyond the next calendar day
3. Hb less than 100 g/L at any time during the preceding 24 hours
4. Age 18 years or greater

## Exclusion criteria

1. Suspected or confirmed severe sepsis (two or more Systemic Inflammatory Response Syndrome (SIRS) criteria, suspected or confirmed infection, and one or more organ system failure)
2. Serum ferritin greater than 1200ng/ml or transferrin saturation greater than 50%
3. History of haemochromatosis or aceruloplasminaemia
4. Known prior administration of IV iron in the preceding 3 months
5. Jehovah’s Witness or other documented exclusion to receiving blood products
6. Receiving ESA (e.g. epoetin or darbepoeitin) in the 3 months prior to ICU admission
7. Known hypersensitivity to intravenous iron
8. Pregnancy
9. Treatment intent is palliative
10. Death is deemed imminent and inevitable
11. Weight less than 40kg
12. Participating in competing study

2. Correlation between hepcidin and other baseline values

Hb -0.09, P=0.308. Ferritin 0.13, P=128. Transferrin saturation -0.15, P=0.09. Soluble transferrin receptor -0.15, P=0.10. C reactive protein -0.09, P= 0.308.

3. Table S1. Iron indices according to hepcidin levels

|  | lowest two hepcidin tertiles | Highest hepcidin tertile | P value |
| --- | --- | --- | --- |
| Iron | 3 (2-7) | 3 (2-6) | 0.363 |
| Transferrin saturation | 9 (6-17) | 8 (6-16) | 0.649 |
| Ferritin | 247 (152-424) | 263 (177-463) | 0.291 |
| Soluble transferrin receptors | 1.82 (1.34-2.54) | 1.63 (1.24-2.13) | 0.225 |
| C reactive Protein | 110 (63-170) | 70 (36-150) | 0.100 |

Median and interquartile range unless otherwise specified

4. Table S2. Univariate analysis of variables associated with risk of RBC transfusion

| Characteristic*  (n=133) | Coefficient (95%CI) | P Value |
| --- | --- | --- |
| **Age** | **-0.013 (-0.026 - -0.002)** | **0.027** |
| Male gender | 0.148 (-0.401 – 0.697) | 0.530 |
| **ICU admission type – trauma vs non trauma** | **0.997 (0.508 – 1.487)** | **<0.001** |
| APACHE II score | -0.001 (-0.40 – 0.038) | 0.954 |
| **SOFA Score** | **0.051 (-0.021 – 0.124)** | **0.165** |
| Renal replacement therapy | 0.382 (-0.665 – 1.429) | 0.475 |
| **Prior RBC transfusion** | **0.571 (-0.008 – 1.149)** | **0.053** |
| **Haemoglobin** | **-0.016 (-0.038 – 0.006)** | **0.145** |
| Mean corpuscular volume | 0.003 (-0.039 – 0.045) | 0.901 |
| C Reactive protein | 0 (-0.003 – 0.002) | 0.823 |
| **Iron** | **0.061 (0.007 – 0.115)** | **0.027** |
| **Ferritin** | **0.001 (-0.0 – 0.002)** | **0.257** |
| **Transferrin** | **-0.043 (-0.085 - -0.0)** | **0.049** |
| **Transferrin saturation** | **0.031 (0.010 – 0.051)** | **0.003** |
| Soluble transferrin receptor | -0.013 (-0.163 – 0.136) | 0.860 |
| Thomas plot (soluble transferrin receptor/log ferritin) | -0.183 (-1.028-0.663) | 0.672 |
| **Hepcidin (mcg/L)** | **0.003 (-0.003 – 0.010)** | **0.286** |
| **Received IV iron** | **-0.338** | **0.188** |

CI confidence interval, ICU intensive care unit, APACHE acute physiology and chronic health evaluation, SOFA sequential organ failure assessment. Variables in bold added to the initial multivariable model.

Figure S1 Relationship between Hepcidin concentration and RBC transfusion moderated by haemoglobin concentration


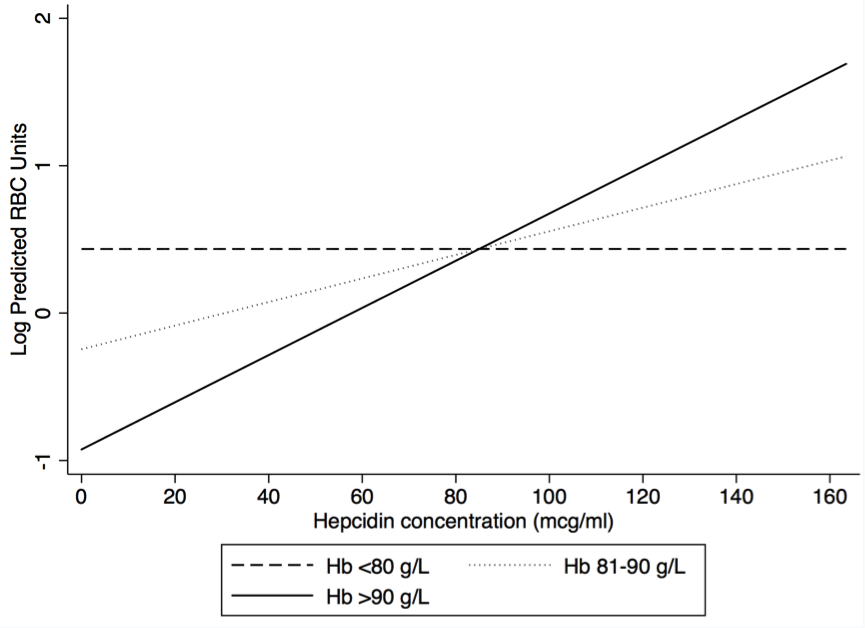


Hb Haemoglobin, RBC red blood cell
